# Heterogeneity in Systematic Reviews of Medical Imaging Diagnostic Test Accuracy Studies: A Systematic Review

**DOI:** 10.1001/jamanetworkopen.2024.0649

**Published:** 2024-02-29

**Authors:** Samuel J. White, Qi Sheng Phua, Lucy Lu, Kaspar L. Yaxley, Matthew D. F. McInnes, Minh-Son To

**Affiliations:** 1 Adelaide Medical School Faculty of Health and Medical Sciences, University of Adelaide, Adelaide, South Australia, Australia; 2 College of Medicine and Public Health, Flinders University, Bedford Park, South Australia, Australia; 3 South Australia Medical Imaging, Flinders Medical Centre, Bedford Park, South Australia, Australia; 4 Department of Radiology, Ottawa Hospital Research Institute, University of Ottawa, Ottawa, Ontario, Canada; 5 Clinical Epidemiology Program, Ottawa Hospital Research Institute, University of Ottawa, Ottawa, Ontario, Canada

## Abstract

**Importance:**

Systematic reviews of medical imaging diagnostic test accuracy (DTA) studies are affected by between-study heterogeneity due to a range of factors. Failure to appropriately assess the extent and causes of heterogeneity compromises the interpretability of systematic review findings.

**Objective:**

To assess how heterogeneity has been examined in medical imaging DTA studies.

**Evidence Review:**

The PubMed database was searched for systematic reviews of medical imaging DTA studies that performed a meta-analysis. The search was limited to the 40 journals with highest impact factor in the *radiology, nuclear medicine, and medical imaging* category in the InCites Journal Citation Reports of 2021 to reach a sample size of 200 to 300 included studies. Descriptive analysis was performed to characterize the imaging modality, target condition, type of meta-analysis model used, strategies for evaluating heterogeneity, and sources of heterogeneity identified. Multivariable logistic regression was performed to assess whether any factors were associated with at least 1 source of heterogeneity being identified in the included meta-analyses. Methodological quality evaluation was not performed. Data analysis occurred from October to December 2022.

**Findings:**

A total of 242 meta-analyses involving a median (range) of 987 (119-441 510) patients across a diverse range of disease categories and imaging modalities were included. The extent of heterogeneity was adequately described (ie, whether it was absent, low, moderate, or high) in 220 studies (91%) and was most commonly assessed using the *I*^2^ statistic (185 studies [76%]) and forest plots (181 studies [75%]). Heterogeneity was rated as moderate to high in 191 studies (79%). Of all included meta-analyses, 122 (50%) performed subgroup analysis and 87 (36%) performed meta-regression. Of the 242 studies assessed, 189 (78%) included 10 or more primary studies. Of these 189 studies, 60 (32%) did not perform meta-regression or subgroup analysis. Reasons for being unable to investigate sources of heterogeneity included inadequate reporting of primary study characteristics and a low number of included primary studies. Use of meta-regression was associated with identification of at least 1 source of variability (odds ratio, 1.90; 95% CI, 1.11-3.23; *P* = .02).

**Conclusions and Relevance:**

In this systematic review of assessment of heterogeneity in medical imaging DTA meta-analyses, most meta-analyses were impacted by a moderate to high level of heterogeneity, presenting interpretive challenges. These findings suggest that, despite the development and availability of more rigorous statistical models, heterogeneity appeared to be incomplete, inconsistently evaluated, or methodologically questionable in many cases, which lessened the interpretability of the analyses performed; comprehensive heterogeneity assessment should be addressed at the author level by improving personal familiarity with appropriate statistical methodology for assessing heterogeneity and involving biostatisticians and epidemiologists in study design, as well as at the editorial level, by mandating adherence to methodologic standards in primary DTA studies and DTA meta-analyses.

## Key Points

**Question:**

How has heterogeneity been assessed in systematic reviews of medical imaging diagnostic test accuracy (DTA) studies?

**Findings:**

This systematic-review of 242 systematic reviews that included studies with a diverse range of disease categories and imaging modalities found that 191 studies (79%) reported moderate to high heterogeneity. However, despite development of more rigorous statistical methods for assessment of heterogeneity, suboptimal or inappropriate statistical methodology was often being used.

**Meaning:**

These findings suggest that inadequate assessment of heterogeneity is compromising the interpretability of many systematic reviews of medical imaging DTA studies.

## Teaser

This systematic review assesses how heterogeneity is examined in systematic reviews of medical imaging diagnostic test accuracy studies.

## Introduction

A rigorous systematic review provides a meticulous summary of available primary research on a given topic, and is underpinned by features such as clear objectives, definition of reproducible methodology a priori and systematic presentation of findings.^1^ These characteristics serve to minimize bias and thereby enhance the credibility of conclusions drawn from the review findings.^1,2^ Systematic reviews of diagnostic test accuracy (DTA) studies aim to provide a complete interpretation of existing data regarding diagnostic test performance in a clear and concise way that has obvious relevance to clinical practice.^3^

The component primary studies brought together for systematic review or meta-analysis typically inherently differ. Although the results of component primary studies will always differ to some degree, heterogeneity or between-study variability describe differences in underlying study parameters^4^ and can be further classified into 3 main subtypes. Clinical heterogeneity refers to differences in the study population, intervention, diagnostic test applied, or outcomes assessed.^2,5^ Methodological heterogeneity refers to heterogeneity in study design and risk of bias.^2,5^ Statistical heterogeneity refers to differences in the measured effect size of an intervention or accuracy of a diagnostic test beyond what would be expected to be attributable to random, within-study variability alone.^5^ Statistical heterogeneity is the product of clinical and methodological variability and randomly occurring effect-size modifiers and only becomes apparent after analysis of the results.^5^ DTA reviews are more likely to be affected by high levels of heterogeneity than reviews of interventions.^6,7^ Reasons for this difference include the common presence of threshold effects in DTA studies,^6^ the tendency for there to be greater differences in clinical parameters (eg, study design, patient population, and test protocol^7,8^) between DTA studies, as well as there being few randomized clinical trials in DTA evaluation despite the ability of randomized clinical trials to confer more thorough control of other variables compared with the observational study designs that are more commonly adopted in DTA studies. Additionally, diagnostic performance is often expressed as a pair of accuracy measures (eg, sensitivity and specificity), whose inherent negative correlation should be accounted for in analyses.^9,10^

Sources of heterogeneity in DTA meta-analyses include between-study differences in patient cohorts, clinical settings, the specific index test used, test interpretation, test positivity threshold, and reference standard.^3,11^ With medical imaging specifically, heterogeneity may include differences in the imaging protocol and sequences (eg, use of biparametric compared with multiparametric magnetic resonance imaging [MRI] for diagnosing prostate cancer^12^), amount of clinical information provided to reporting radiologists,^13^ sonographer experience,^14^ evolution of imaging technology over time (eg, 3 T compared to 1.5 T MRI^15^), and benchmark imaging modality or other test being used as the reference standard (eg, histopathology-based reference standards compared with clinical reference standards in studies examining the accuracy of liver imaging reporting and data systems for hepatocellular carcinoma^16^).

When a random-effects model (eAppendix 1 in Supplement 1) is incorporated in DTA meta-analyses (as recommended by methodological guidance^17^), estimated effect sizes will represent the mean of a distribution of effect sizes across a population of studies with different true effect sizes. If these true effect sizes are very different from each other, it may not be meaningful to combine them in a meta-analysis without giving due consideration to sources of heterogeneity. The presence of such heterogeneity (especially if unaccounted for) can make it difficult to form robust conclusions regarding test performance in clinical practice.

Dinnes and colleagues^8^ performed a systematic review describing the assessment of heterogeneity in DTA studies published prior to December 2002. Importantly, the bivariate model, which is now one of the most commonly used methods for DTA meta-analyses, was first proposed in 2005.^18^ The assessment of heterogeneity in medical imaging DTA systematic reviews has, to our knowledge, not previously been evaluated; this is important considering the unique sources of heterogeneity in medical imaging DTA studies that are not otherwise present in other DTA studies, as well as the key role medical imaging DTA studies play in informing guidelines and standard-of-care measures. We conducted a systematic review to determine how heterogeneity has been examined in medical imaging DTA meta-analyses.

## Methods

This systematic review followed the Preferred Reporting Items for Systematic Reviews and Meta-Analyses (PRISMA) reporting guideline^19^ and did not require institutional review board approval because it did not constitute human participants research in accordance with the Common Rule. The study protocol was prepared a priori and is available on the Open Science Framework.^20^

### Search Strategy

The InCites Journal Citation Reports of 2021^21^ were used to identify the 40 journals with highest impact factor in the *radiology, nuclear medicine, and medical imaging* category to reach a sample size of 200 to 300 included studies. Journals were searched in declining order of impact factor until the target range of studies was reached. Once the target range of studies was reached, we rounded the number of journals to the nearest 10, resulting in 40 journals being included in our search. The search string (eAppendix 2 in Supplement 1) was used to search the PubMed database on October 4, 2022, to find systematic reviews of diagnostic imaging accuracy studies that performed meta-analyses published in those 40 journals between January 1, 2005, and December 31, 2021. We did not consider it essential to systematically identify all available medical imaging DTA reviews for the purposes of this methodological study. The starting date was selected due to the development of the bivariate approach to performing meta-analyses of diagnostic accuracy data in 2005^18^ (and, thus, there was increasing use of this model from this year onwards).

### Eligibility Criteria

Systematic reviews of diagnostic imaging accuracy studies that performed meta-analysis were included. We defined systematic reviews as reviews that supplied evidence of a search strategy defined a priori that was sufficiently detailed to allow replication of the search. The exclusion criteria were as follows: no meta-analysis performed; the study was a cost analysis or network analysis; the study was of predictive or prognostic tests or of individual patient data, animal studies, and non-English studies. Studies were not excluded on the basis of whether they used the bivariate model. Screening of studies was performed independently by 2 authors (L.L. and Q.S.P.), and disagreements were resolved by a third author (M.S.T.).

### Data Extraction

Data collection from included full-text studies was independently performed by 2 authors (S.W. and Q.S.P.) using the data extraction template shown in eAppendix 3 in Supplement 1. Comparative design studies (eg, MRI vs computed tomography) were classified according to the first listed imaging modality in the title. Conflicts were resolved by a third author (M.S.T.).

### Statistical Analysis

Regression analysis was performed using RStudio version 1.4.1106 (Posit) for MacOS (Apple). We dichotomized meta-analyses by whether they identified at least 1 specific source of variability, and then used multivariable logistic regression to study associations of characteristics of the included meta-analyses with this dichotomy. Handling of missing data are described in eAppendix 4 in Supplement 1. Statistical significance was considered a 2-sided *P* < .05. Data analysis occurred from October to December 2022.

## Results

The literature search yielded 743 results (Figure). After screening, 305 full-text studies were assessed for eligibility, with 242 studies being included in this review. Included full-text articles are available on the Open Science Framework.^20^

**Figure.**
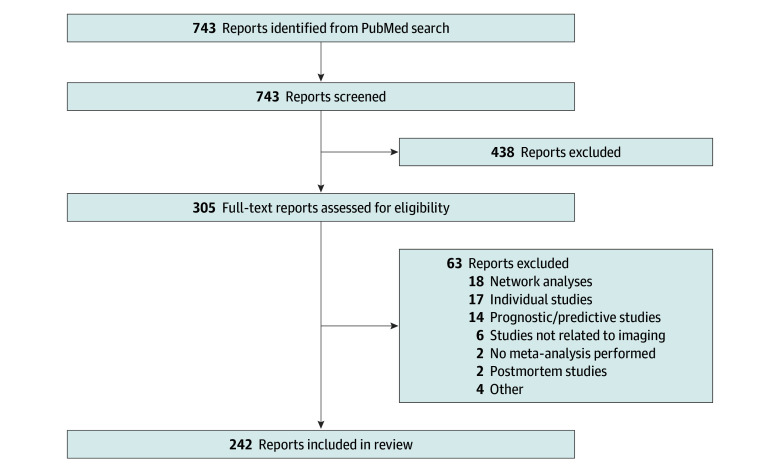
Flow Diagram of Literature Search and Screening

The median (range) year of publication was 2017 (2005-2022) (Table 1). *European Radiology* was the most common journal in which included studies were published (67 studies [28%]). The median (range) 2023 impact factor (4.6 [1.0-29.2]) was calculated by assigning each of the 242 included studies an impact factor. The most common disease category assessed was gastrointestinal (52 studies [21%]), and the most common imaging modality was MRI (92 studies [38%]). The median (range) number of component primary studies included in the meta-analyses was 14 (4-117). The median (range) number of patients per meta-analysis was 987 (119-441 510). The median (range) time between earliest and latest component studies included in a meta-analysis was 10 (1-41) years.

**Table 1.** Characteristics of Included Meta-Analyses

Characteristic	Studies, No. (%) (N = 242)
Year of publication, median (range)	2017 (2005-2022)
No. of component primary studies, median (range)	14 (4-117)
No. of patients per meta-analysis, median (range)	987 (119-441 510)
Imaging modality	
Computed tomography	63 (26)
Magnetic resonance imaging	92 (38)
Ultrasonography	31 (13)
Positron emission tomography	39 (16)
Other	17 (7)
Disease category	
Breast	14 (6)
Cardiovascular	38 (16)
Thoracic	16 (7)
Gastrointestinal	52 (21)
Genitourinary	39 (16)
Head and neck	10 (4)
Musculoskeletal	24 (10)
Neurological	31 (13)
Pediatric	6 (2)
Other	12 (5)

Meta-analysis was performed using a bivariate model in 145 studies (60%) (Table 2). A univariate model was used in 53 studies (22%), a hierarchical summary receiver operating characteristic (HSROC) model alone was used in 7 studies (3%), and the statistical methodology was unclear in 37 studies (15%). Reviews that used an HSROC model and either a bivariate or a univariate model were classified as either bivariate or univariate. Among the 145 studies that used a bivariate model, 114 (79%) constructed a SROC curve. The terms *heterogeneity* or *variability* were explicitly mentioned in 234 studies (97%) and was assessed using at least 1 statistical test in 208 studies (86%). Of all 242 meta-analyses, 185 (76%) assessed heterogeneity using *I*^2^; of these 185 studies, 63 (34%) used a univariate model and 122 (66%) used the bivariate model. Heterogeneity was depicted using a forest plot in 181 studies (75%) and SROC curves in 184 studies (76%). Pooled sensitivity and specificity was performed in 227 studies (95%), and a pooled likelihood ratio was provided in 97 studies (41%) (eTable 1 in Supplement 1). Threshold effects were considered in 80 studies (33%).

**Table 2.** Summary of Results

Result	Studies, No. (%) (N = 242)
Type of model	
Hierarchic summary receiver operating characteristic only	7 (3)
Bivariate	145 (60)
Univariate	53 (22)
Not stated or unclear	37 (15)
Heterogeneity explicitly mentioned	234 (97)
Test used to assess heterogeneity	
* I^2^* statistic	185 (76)
Cochran* Q* statistic	92 (38)
χ^2^ statistic	63 (26)
Spearman correlation coefficient	57 (24)
Forest plot	181 (75)
Other	74 (31)
Consideration of threshold effects	80 (33)
Subjective description of level of heterogeneity	
Low or none	29 (12)
Moderate	80 (33)
High	111 (46)
Unclear	22 (9)
Identification of sources of heterogeneity	
Performed subgroup analysis	122 (50)
Performed meta-regression	87 (36)
Sources of heterogeneity identified	
Clinical	40 (17)
Socioeconomic	1 (<1)
Test-related	60 (25)
Threshold-related	27 (11)
Quality-related	17 (7)
Other	53 (22)

The extent of statistical heterogeneity between component primary studies included in meta-analyses was described as low or absent in 29 studies (12%), present or moderate in 80 studies (33%), and high in 111 studies (46%). The level of heterogeneity was not stated or not assessed in 22 studies (9%). Overall, heterogeneity was rated as moderate or high in a substantial number of studies (191 studies [79%]). A few studies referred to *I^2^* to support their classification of the extent of heterogeneity. Explanation of how heterogeneity was classified was seldom provided.

In terms of identifying statistically significant sources of heterogeneity, 122 meta-analyses (50%) performed subgroup analysis and 87 (36%) performed meta-regression. Of the 242 studies assessed, 189 (78%) included 10 or more primary studies. Of these 189 studies, 60 (32%) did not perform meta-regression or subgroup analysis. Only 13 of 160 studies that performed subgroup analysis or meta-regression (8.1%) planned this a priori. Among the 87 studies that performed meta-regression, the median (range) of included studies was 16 (5-88) studies, and 17 of these studies (20%) included less than 10 primary studies. The median (range) number of variables assessed per model was 6 (1-21). At least 1 source of heterogeneity was identified in 53 of 87 studies that performed meta-regression (61%). In all 242 studies, heterogeneity was adequately described (ie, whether it was absent, low, moderate, or high) in 220 studies (91%). Heterogeneity was attributed to differences in clinical factors in 40 studies (17%), socioeconomic factors in 1 study (<1%), test-related factors in 60 studies (25%), threshold-related factors in 27 studies (11%), quality-related factors (such as patient enrollment^22,23^ and blinding^22^) in 17 studies (7%), and other factors in 53 studies (22%). Examples of other sources of heterogeneity included the level of experience and specialization of the individual reviewing imaging,^23^ type of image analysis performed (quantitative vs qualitative),^24^ and study type (prospective vs retrospective).^25^ Most of the meta-analyses that did not investigate potential sources of heterogeneity provided no specific justification for that omission, but several cited inadequate reporting of component study characteristics^26,27,28^ and several others mentioned insufficient numbers of included primary studies.^29,30,31,32^ The median number of included studies in reviews that did not investigate potential sources of heterogeneity was 13 studies compared with 15 for reviews that did.

Multivariable logistic regression was performed to assess factors associated with at least 1 source of heterogeneity being identified in the included meta-analyses (eTable 2 in Supplement 1); factors included year of publication, journal impact factor, number of component primary studies, total number of patients included in the meta-analysis, whether subgroup analysis was performed, and whether meta-regression was performed. Data for the total number of patients was missing for 15 of the 242 included studies (6.2%) (eAppendix 4 in Supplement 1). Use of meta-regression analysis was associated with identification of at least 1 source of variability (odds ratio, 1.90; 95% CI, 1.11-3.23; *P* = .02), whereas there was no association with subgroup analysis (odds ratio, 1.72; 95% CI, 1.02-2.83; *P* = .05). Year of publication, journal impact factor, number of component primary studies, and total number of patients were not statistically significantly associated with whether at least 1 source of heterogeneity was identified in the included meta-analyses. Based on our findings, we developed a list of key recommendations for estimating, identifying, and improving reporting of heterogeneity (Table 3).^33,36,37^

**Table 3.** Key Recommendations for Improving Reporting and Assessment of Heterogeneity in DTA Research

Recommendation category	Recommendations
Identifying potential sources of heterogeneity in medical imaging DTA systematic reviews	
1	A priori consideration of heterogeneity sources based on scientifically plausible hypotheses or previous evidence syntheses (eg, patient demographics such as pediatric vs adult patients and threshold effects such as index test positivity criteria); planned investigations of heterogeneity should be published a priori in the study protocol
2	At a minimum, visual inspection of heterogeneity using forest plots (separate plots of sensitivity and specificity), paying particular attention to the proximity of data points to the summary receiver operating characteristic curve and less attention to how scattered the data points are from one another,^3^ is recommended to assess for the presence of heterogeneity prior to deciding whether to statistically pool data
3	Variation of sensitivity and specificity pairs in receiver operating characteristic space (sensitivity vs 1 − specificity)
Measuring heterogeneity in medical imaging DTA systematic reviews	
1	Where there is a sufficient number of primary studies, use of a hierarchical model for subgroup analysis (noting the bivariate model intuitively makes more sense in the context of imaging studies); subgroup analysis should be identified a priori in the study protocol, and additional subgroups should be analyzed only when there is a strong visual signal (eg, from forest plots)
2	Use of prediction regions (ellipses) where data are plotted in receiver operating characteristic space provided the number of primary studies is ≥10^3,33^
3	The *I^2^* statistic may be an appropriate measure of heterogeneity where univariate models are used (ie, where a bivariate model cannot be used due to inestimable sensitivity and specificity [eg, primary studies with 0 denominators or where summary estimates across the 2 models are deemed sufficiently similar])^34,35^
Improving reporting of heterogeneity in primary DTA studies	
1	Prospective registration of primary DTA studies and systematic reviews of DTA studies
2	Mandate adherence to the 2015 Standards for Reporting Diagnostic Accuracy Studies guideline^36^ in primary medical imaging DTA studies
3	Mandate adherence to checklist items regarding heterogeneity assessment (13d, 13e, 20b, and 20c) in the 2020 Preferred Reporting Items for Systematic Reviews and Meta-Analyses guideline^19^ in medical imaging DTA systematic reviews (it should be noted that the current Preferred Reporting Items for Systematic Reviews and Meta-Analyses-DTA extension^37^ does not feature these items)
4	Greater emphasis on heterogeneity assessment in the tertiary education sector
5	Early involvement of biostatisticians and epidemiologists in primary DTA studies and DTA systematic reviews
6	Improve personal familiarity with contemporary guidance regarding heterogeneity assessment prior to conducting DTA systematic reviews; Lee and colleagues^7^ have published a useful methodological reference article that comprehensively outlines statistical methods recommended for performing meta-analyses of DTA studies

Abbreviation: DTA, diagnostic test accuracy.

## Discussion

This systematic review investigated the assessment of heterogeneity in DTA meta-analyses in the medical imaging literature. The search strategy captured a diverse range of disease categories, imaging modalities, and meta-analysis sizes. Heterogeneity was assessed to some extent in the majority of meta-analyses reviewed (220 of 242 studies [91%]) and was most commonly assessed using *I*^2^ and forest plots. The extent of subjective heterogeneity was described as low or absent in just 29 meta-analyses (12%), although methods of classification were seldom described. Inadequate reporting of primary study characteristics and an inadequate number of primary studies were reasons provided for failure to examine sources of heterogeneity. Completion of meta-regression analysis was the only statistically significant association of detection of sources of heterogeneity in the included meta-analyses, although results for subgroup analysis were numerically quite similar. However, 17 of 87 reviews that performed meta-regression (20%) did so using less than 10 primary studies, which is not advisable.^2^

### Assessment of Heterogeneity

A variety of tools were used to assess heterogeneity. Of the 242 studies, 208 (86%) used at least 1 statistical test or descriptive statistic to examine heterogeneity with *I*^2^ being the most popular (185 studies [76%]). In contrast, in their 2005 review, Dinnes et al^8^ investigated assessment of heterogeneity in DTA studies and observed that only 41% of meta-analyses used a statistical test. Increased use of statistical tests for heterogeneity is not necessarily a marker of higher quality analysis, and given the proliferation of heterogeneity in DTA reviews, quantification of heterogeneity is considerably less valuable than investigation of its causes. Tests used to measure heterogeneity are known to have low statistical power.^7,38^ Furthermore, *I*^2^ is often not appropriate for use in systematic reviews of DTA studies (particularly when the bivariate model has been used) because it is a univariate measure that does not account for threshold effects or the expected inverse correlation between sensitivity and specificity, which may contribute to a sizeable proportion of variation between studies. We observed that 122 of 185 studies that used *I*^2^ (66%) used the bivariate model, which represents a substantial proportion of studies adopting inappropriate statistical methodology to estimate heterogeneity. The Cochran* Q* test similarly does not account for threshold effects.^7^ Although *I*^2^ estimates the proportion of total variation attributable to between-study heterogeneity (as opposed to chance), *Τ^2^* is an estimate of the amount of true heterogeneity in results between studies^39^ and is, therefore, probably more useful in the context of DTA meta-analyses. Variances of logit sensitivity and specificity may also provide an appropriate indication of heterogeneity. Sometimes, though, graphical representation of heterogeneity provides clearer insight than use of statistical tests. While our study reported use of a forest plot in 181 meta-analyses (75%), Dinnes et al^8^ observed use of a graphical plot in 56% of studies. The use of prediction ellipses around SROC curves (the approximate bivariate equivalent of a prediction interval)^40^ is another suitable alternative for graphically depicting heterogeneity in DTA meta-analyses. Ultimately, the decision to statistically pool data in a DTA meta-analysis should be based on both clinical and methodological assessment of the differences between studies. Reliance upon a statistical test alone to guide decision-making is inappropriate.

We noted that the majority of studies used hierarchical models (bivariate and/or the HSROC model) for meta-analysis. Such models are increasingly being used in DTA meta-analyses because they incorporate random study effects to account for unexplained heterogeneity, which is highly likely to be encountered among primary DTA studies. Both models also account for the expected negative correlation between sensitivity and specificity that results from varying the explicit or implicit test positivity threshold (as visualized by the SROC curve), which is an additional source of heterogeneity not taken into account by univariate models (these tend to treat sensitivity and specificity as independent parameters and therefore meta-analyze them separately). However, there is an important conceptual difference between the bivariate and HSROC models. The former seeks to compute an average sensitivity and specificity across primary studies included in the meta-analysis, while the latter attempts to fit a summary (average) ROC curve. Despite these differences, the bivariate and HSROC models are mathematically equivalent when no covariates are included in the model specification.^17^ Thus, an SROC curve is commonly derived from the bivariate model output, which was commonly observed in the meta-analyses included in this review. However, it is not clear how to interpret this output. In the case of a numerical index test, such as a laboratory blood test, the interpretation of the SROC curve is more straightforward because the curve shape is dictated by the overall results of individual studies in the meta-analysis, an important component of which is the specific test cut-off value used in individual studies. Imaging tests, however, are usually binary and may involve an element of subjective interpretation. Thus, variation along the SROC curve may represent implicit threshold effects (eg, variation in radiologist internal threshold for test positivity) or apparent threshold effects resulting from other sources of heterogeneity not explicitly modeled. Rarely, however, was an interpretation given for these displayed curves. Supplying a summary sensitivity and specificity value from the bivariate model is also problematic when there is substantial variation across or away from the SROC curve,^17^ although subgroup analysis may partly address this problem.

### Identification of Sources of Heterogeneity

Most meta-analyses included in this review were affected by moderate to high levels of heterogeneity. This is important because even rigorously performed meta-analyses of highly heterogeneous component studies pose interpretive challenges.^41,42^ Identifying specific sources of heterogeneity is therefore critical to informing interpretation and maintaining the clinical utility of meta-analyses of heterogeneous studies. For example, Sonnad and colleagues^43^ performed a meta-analysis examining the accuracy of MRI for staging prostate cancer and observed a maximum joint sensitivity and specificity of 74%. The authors^43^ subsequently performed a subgroup analysis that found fast spin echo imaging was significantly more accurate than conventional spin-echo imaging, suggesting that evolution of imaging technology was a cause of heterogeneity. However, the authors^43^ were unable to perform subgroup analysis based on characteristics of the study population due to inadequate reporting in primary studies. The appropriateness of applying the review’s findings to broad patient populations was therefore unascertainable, limiting the clinical utility of the meta-analysis.^43^

Regression analysis demonstrated that completion of meta-regression analysis was associated with identification of sources of heterogeneity and results for subgroup analysis were numerically quite similar although not statistically significant. Reasons for failure to assess statistically possible sources of heterogeneity included inadequate reporting of component study characteristics^26,27,28^ and insufficient statistical power.^29,30,31,32^ Lack of confidence in using appropriate statistical methods to investigate sources of heterogeneity is another likely contributing factor.

### Recommendations

Interrogating potential sources of heterogeneity in medical imaging DTA reviews may lead to identification of specific patient groups and clinical settings best suited to a given diagnostic imaging modality or protocol, which is more useful to policymakers than a simple common summary estimate of accuracy^44^ Table 3 summarizes our key recommendations with respect to estimating and identifying sources of heterogeneity in medical imaging DTA meta-analyses, as well as improving reporting of heterogeneity more broadly in DTA literature. If it is deemed appropriate to pool data, contemporary guidance recommends meta-regression using study-level covariates in a hierarchical model^17^ as the most efficient statistical strategy for evaluating sources of heterogeneity between DTA studies. This allows any differential effect of a covariate on sensitivity and/or specificity (bivariate model) or accuracy (curve position [HSROC model]) to be explored. Quality assessment domains should be included where possible.^17^ Specific guidance on the minimum number of DTA studies needed before conducting such meta-regression analyses is lacking. However, for intervention studies, meta-regression is not recommended to be performed when there are less than 10 primary studies available.^2^ In general, a balance between the number of studies and number of covariates that can be reasonably investigated is important.

### Limitations

This study had several limitations. The search strategy was specific to the 40 highest impact medical imaging journals.^45^ Therefore, relevant imaging DTA meta-analyses published in other journals may have not been captured, resulting in our cohort of 242 included studies not being fully representative of work published in nonmedical imaging or lower impact factor journals. Second, while this study characterized the assessment of heterogeneity in medical imaging DTA meta-analyses, it did not examine the impact of heterogeneity on study outcomes. When there is substantial between-study variability, studies become weighted nearly equally irrespective of sample size in random-effects meta-analyses, resulting in the meta-analytic summary estimate moving closer to the arithmetic mean of the individual study results.^17,41,46,47,48^ This means that studies with small sample sizes have a disproportionate impact on the summary estimate. Because small study effects have widespread prevalence in the medical imaging DTA literature^49^ and because publication bias favors studies reporting more positive conclusions,^50^ it follows that meta-analyses of heterogeneous studies are susceptible to inflated estimates of imaging test performance.

## Conclusions

In this systematic review of assessment of heterogeneity in medical imaging DTA meta-analyses, heterogeneity was recognized, and, to some extent, described in the majority of included medical imaging DTA meta-analyses, although some studies used inappropriate statistical methodology. Most studies were affected by moderate to high levels of heterogeneity, and completion of meta-regression was shown to be associated with identification of sources of heterogeneity. However, meta-regression is not appropriate in some contexts. Medical imaging DTA studies play a critical role in informing development of radiological guidelines and standard-of-care measures. It is, therefore, imperative to not only quantify the level of heterogeneity using appropriate statistical and/or graphical methodology, but also identify underlying sources when possible. This aids interpretation of meta-analyses of medical imaging DTA studies and informs the appropriateness of applying results to broader clinical contexts.

## Notes

Supplement 1. **eAppendix 1.** Summary of Random Effects Models Assessed in the Context of Diagnostic Test Accuracy Systematic Reviews
eReferences
**eAppendix 2.** Complete Search String Used for PubMed Database Search **eAppendix 3.** Data Extraction Template **eAppendix 4.** Handling of Missing Data **eTable 1.** Cross-Tabulation of Statistical Methodology Used and Summary Metrics Reported **eTable 2.** Multivariable Logistic Regression Output for Factors Predictive of at Least One Source of Heterogeneity Being Identified Amongst Included Meta-Analyses

Supplement 2.
Data Sharing Statement
