# Supplementary material for: Heterogeneity in Systematic Reviews of Medical Imaging Diagnostic Test Accuracy Studies: A Systematic Review
Source: JAMA Netw Open. 2024 Feb 29;7(2):e240649. doi: 10.1001/jamanetworkopen.2024.0649 (PMC10905313; doi:10.1001/jamanetworkopen.2024.0649)
Supplement: Supplement 1. — eAppendix 1. Summary of Random Effects Models Assessed in the Context of Diagnostic Test Accuracy Systematic Reviews eReferences eAppendix 2. Complete Search String Used for PubMed Database Search eAppendix 3. Data Extraction Template eAppendix 4. Handling of Missing Data eTable 1. Cross-Tabulation of Statistical Methodology Used and Summary Metrics Reported eTable 2. Multivariable Logistic Regression Output for Factors Predictive of at Least One Source of Heterogeneity Being Identified Amongst Included Meta-Analyses [file jamanetwopen-e240649-s001.pdf]

Supplemental Online Content

White SJ, Phua QS, Lu L, Yaxley KL, McInnes MDF, To MS. Heterogeneity in systematic reviews of medical imaging, diagnostic test accuracy studies: a systematic review. *JAMA Netw. Open.* 2023;7(2):e240649.
doi:10.1001/jamanetworkopen.2024.0649

eAppendix 1. Summary of Random Effects Models Assessed in the Context of Diagnostic Test Accuracy Systematic Reviews
eReferences
eAppendix 2. Complete Search String Used for PubMed Database Search
eAppendix 3. Data Extraction Template
eAppendix 4. Handling of Missing Data
eTable 1. Cross-Tabulation of Statistical Methodology Used and Summary Metrics Reported
eTable 2. Multivariable Logistic Regression Output for Factors Predictive of at Least One Source of Heterogeneity Being Identified Amongst Included Meta-Analyses

This supplemental material has been provided by the authors to give readers additional information about their work.

© 2024 White SJ et al. *JAMA Network Open.*

eAppendix 1. Summary of Random Effects Models Assessed in the Context of Diagnostic Test Accuracy Systematic Reviews

Univariate model: Performs meta-analysis of diagnostic test accuracy by separate pooling of sensitivity and specificity, using a fixed or random effects model. This generates summary estimates of sensitivity and specificity analogous to the pooled effect estimates obtained in meta-analyses of therapeutic studies[1]. However, this approach does not take into account any correlation between these parameters (an inverse relationship between sensitivity and specificity is expected, for instance, as the test positivity threshold varies).

Hierarchical model: allow joint modelling of sensitivity and specificity, taking into account any correlation between them. The modelling is performed at two levels; modelling of the cell counts in the extracted 2x2 tables from primary studies using binomial distributions (the lower level) and modelling of the correlation between sensitivity and specificity (using additional parameters; the higher level)[2]. Two methods are available: the bivariate model and the hierarchical summary receiver operating characteristic (HSROC) model. Both formulations have been shown to be different parameterisations of the same model, provided no covariates are included[3] but differ in their conceptual approach.

Bivariate model: generates a summary estimate of sensitivity and specificity (a summary point) across all studies. Intuitively, the model is appropriate if it is expected there is a common underlying test threshold among the primary studies in the analysis, and thus reporting of the 'average' level of diagnostic accuracy across all studies is preferred.

© 2024 White SJ et al. *JAMA Network Open.*

HSROC model: generates a summary ROC curve (SROC). Conceptually, this is an ROC curve averaged across studies and provides visual representation of how sensitivity and specificity vary as the test threshold varies. It is appropriate if there is felt to be significant threshold variation across studies. Each study is plotted as a single sensitivity, specificity pair and the model fits a curve across the data. The model includes parameters that determine the position and shape of the curve. This method also allows qualitative evaluation of heterogeneity as variability away from the SROC curve would be expected to represent greater heterogeneity than variation along the SROC curve (the latter may just correspond to threshold effects). Given the bivariate and HSROC models are mathematically equivalent, the bivariate model can be used to generate an SROC curve. This is a reasonable approach (and commonly performed) as summary points and SROC curves may provide complementary information[2].

eReferences

1. Lee J, Kim KW, Choi SH, Huh J, Park SH. Systematic Review and Meta-Analysis of Studies Evaluating Diagnostic Test Accuracy: A Practical Review for Clinical Researchers-Part II. Statistical Methods of Meta-Analysis. *Korean J Radiol.* 2015;16(6):1188-1196.
2. Macaskill P, Takwoingi Y, Deeks JJ, Gatsonis C. Chapter 9: Understanding meta-analysis. In: Deeks JJ, Bossuyt PM, Leeflang MM, Takwoingi Y, eds. *Cochrane Handbook for Systematic Reviews of Diagnostic Test Accuracy (Version 2.0).* Cochrane; 2023.
3. Harbord RM, Deeks JJ, Egger M, Whiting P, Sterne JA. A unification of models for meta-analysis of diagnostic accuracy studies. *Biostatistics.* 2007;8(2):239-251.

© 2024 White SJ et al. *JAMA Network Open.*

eAppendix 2. Complete Search String Used for PubMed Database Search

((meta-analysis[Title/Abstract]) OR (meta-analysis as topic[MeSH Terms]) OR(meta-analysis[Publication Type])) AND ((RADIOLOGY[Journal]) OR (JACC-Cardiovascular Imaging[Journal]) OR (Medical Image Analysis[Journal]) OR (Journal of Nuclear Medicine[Journal]) OR (IEEE TRANSACTIONS ON MEDICAL IMAGING[Journal]) OR (Clinical Nuclear Medicine[Journal]) OR (INVESTIGATIVE RADIOLOGY[Journal]) OR (EUROPEAN JOURNAL OF NUCLEAR MEDICINE AND MOLECULAR IMAGING[Journal]) OR (Photoacoustics[Journal]) OR (European Heart Journal-Cardiovascular Imaging[Journal]) OR (ULTRASOUND IN OBSTETRICS AND GYNECOLOGY[Journal]) OR (Circulation-Cardiovascular Imaging[Journal]) OR (INTERNATIONAL JOURNAL OF RADIATION ONCOLOGY BIOLOGY PHYSICS[Journal]) OR (COMPUTERIZED MEDICAL IMAGING AND GRAPHICS[JOURNAL]) OR (NEUROIMAGE[Journal]) OR (Diagnostic and Interventional Imaging[Journal]) OR (Zeitschrift fur Medizinische Physik[Journal]) OR (KOREAN JOURNAL OF RADIOLOGY[Journal]) OR (EUROPEAN RADIOLOGY[Journal]) OR (JOURNAL OF CARDIOVASCULAR MAGNETIC RESONANCE[Journal]) OR (RADIOGRAPHICS[Journal]) OR (RADIOTHERAPY AND ONCOLOGY[Journal]) OR (HUMAN BRAIN MAPPING[Journal]) OR (AMERICAN JOURNAL OF ROENTGENOLOGY[Journal]) OR (Journal of the American College of Radiology[Journal]) OR (SEMINARS IN RADIATION ONCOLOGY[Journal]) OR (Cancer Imaging[Journal]) OR (Journal of Thoracic Imaging[Journal]) OR (Academic Radiology[Journal]) OR (JOURNAL OF MAGNETIC RESONANCE IMAGING[Journal]) OR (AMERICAN JOURNAL OF NEURORADIOLOGY[Journal]) OR (JOURNAL OF DIGITAL IMAGING[Journal]) OR (Insights into Imaging[Journal]) OR (SEMINARS IN NUCLEAR MEDICINE[Journal]) OR (Ultrasonography[Journal]) OR (EJNMMI Physics[Journal]) OR (Clinical and Translational Radiation Oncology[Journal]) OR (Physical and Engineering Sciences in Medicine [Journal]) OR (Radiologia Medica[Journal]) OR (ULTRASCHALL IN DER MEDIZIN[Journal])) AND ((diagnostic test accuracy OR DTA [all fields]) OR (sensitivity and specificity [all fields]) OR (sensitiv*[all fields]) OR(specificit*[all fields]) OR (accuracy[all fields]) OR (ROC [all fields]) OR (“receiver operator characteristic” [all fields]) OR (“diagnostic odds ratio” [all fields]) OR (“likelihood ratio” [all fields]) OR (“positive predictive value” [all fields]) OR (PPV [all fields]) OR (“negative predictive value”[all fields]) OR (NPV[all fields]) OR (false positive*[all fields]) OR (false negative*[all fields])) AND (2005/01/01[PDat]:2021/12/31[PDat])

© 2024 White SJ et al. *JAMA Network Open.*

eAppendix 3. Data Extraction Template

Basic information
Lead author of study: ______________________
Year of publication: ______________________
Journal: ______________________
Journal impact factor: ______________________
Statistician, epidemiologist or public health physician listed as co-author?: __________________
Use of PRISMA checklist or similar tool to conduct systematic review?: ____________________

Meta-analysis size
Number of component primary studies: ______________________
Total number of patients: ______________________

Subject of meta-analysis
Imaging modality: ______________________
Disease tested for:

- Breast
- Cardiovascular
- Thoracic
- Gastrointestinal
- Genitourinary
- Head and neck
- Musculoskeletal
- Neurological
- Paediatric
- Other

Quality of meta-analysis
Type of meta-analysis model:

- Summary ROC
- Hierarchical SROC
- Univariate
- Bivariate random-effects
- Other: ______________________

Time since first publication at time of review publication (years): ______________________
Time between first and last publication (years): ______________________

Test for heterogeneity
Is heterogeneity or variability mentioned?: ______________________
How is heterogeneity tested for?

- i^2:
- Cochrane's Q statistic:
- Chi-square:
- Spearman correlation coefficient:
- Forest plot:
- Other: ______________________

Was there pooled sensitivity/specificity?: ______________________
Was there pooled likelihood ratio?: ______________________
Was there summary ROC curve?: ______________________
Was there regression?: ______________________
Other test?: ______________________
Software model: ______________________

Identification of sources of heterogeneity
Was subgroup analysis done?: ______________________
Was meta-regression done?: ______________________

© 2024 White SJ et al. *JAMA Network Open.*

Other test done?: ____________________

Sources of varia heterogeneity bility
- Clinical: ____________________
- Socioeconomic: ____________________
- Test-related: ____________________
- Threshold-related: ____________________
- Quality-related: ____________________
- Other: ____________________

eAppendix 4. Handling of Missing Data

The randomness of missing data was assessed by using multivariable logistic regression to regress each variable onto the column with missing data. This analysis did not yield a statistically significant result ($p > 0.05$), indicating data was missing completely at random. We performed multiple imputation using predictive mean matching because the missing variable (number of patients) was a continuous variable.

eTable 1. Cross-Tabulation of Statistical Methodology Used and Summary Metrics Reported

Statistical methodology	Pooled sensitivity and specificity	Pooled likelihood ratio
Bivariate model	137/145 (94%)	54/145 (37%)
Univariate model	51/53 (96%)	29/53 (55%)
HSROC alone	4/7 (57%)	1/7 (14%)
Unclear methodology	35/36 (97%)	13/36 (36%)

HSROC: hierarchical summary receiver operating characteristic

© 2024 White SJ et al. *JAMA Network Open.*

eTable 2. Multivariable Logistic Regression Output for Factors Predictive of at Least One Source of Heterogeneity Being Identified Amongst Included Meta-Analyses

	**Estimated parameter**	**Standard error**	**Z-value**	**P-value**
(Intercept)	-26.6	66.3	-0.40	0.69
Year of publication (per decade)	0.12	0.33	0.39	0.70
Journal impact factor (per 100 impact factor)	0.44	1.6	0.28	0.78
Number of component primary studies (per 100 primaury studies)	0.17	0.90	0.19	0.85
Total number of patients (per 100,000 patients)	-0.54	0.82	-0.65	0.52
Subgroup analysis performed	0.54	0.28	1.93	0.053
Meta-regression performed	0.64	0.28	2.27	0.023*

* $p < 0.05$

© 2024 White SJ et al. *JAMA Network Open.*
