# Supplementary material for: Heterogeneity in Systematic Reviews of Medical Imaging Diagnostic Test Accuracy Studies: A Systematic Review
Source: JAMA Netw Open. 2024 Feb 29;7(2):e240649. doi: 10.1001/jamanetworkopen.2024.0649 (PMC10905313; doi:10.1001/jamanetworkopen.2024.0649)
Supplement: Supplement 2. — Data Sharing Statement [file jamanetwopen-e240649-s002.pdf]

Data Sharing Statement

White. Heterogeneity in Meta-Analyses of Medical Imaging, Diagnostic Test Accuracy Studies. *JAMA Netw Open*. Published February 29, 2024. doi:10.1001/jamanetworkopen.2024.0649

Data
Data available: No

Additional Information
Explanation for why data not available: This is a meta-research study and therefore data is already publicly available through online databases
